# Supplementary material for: Differential Requirements for the RAD51 Paralogs in Genome Repair and Maintenance in Human Cells
Source: PLoS Genet. 2019 Oct 4;15(10):e1008355. doi: 10.1371/journal.pgen.1008355 (PMC6795472; doi:10.1371/journal.pgen.1008355)
Supplement: S1 References — (DOCX) [file pgen.1008355.s001.docx]

**S1 References.** Supporting information references.

23. Schild D, Lio YC, Collins DW, Tsomondo T, Chen DJ. Evidence for simultaneous protein interactions between human Rad51 paralogs. J Biol Chem. 2000;275: 16443–16449. doi:10.1074/jbc.M001473200

81. Feng W, Jasin M. BRCA2 suppresses replication stress-induced mitotic and G1 abnormalities through homologous recombination. Nat Commun. 2017;8: 525. doi:10.1038/s41467-017-00634-0

83. Hockemeyer D, Soldner F, Beard C, Gao Q, Mitalipova M, DeKelver RC, et al. Efficient targeting of expressed and silent genes in human ESCs and iPSCs using zinc-finger nucleases. Nat Biotechnol. 2009;27: 851–857. doi:10.1038/nbt.1562

103. Kondrashova O, Nguyen M, Shield-Artin K, Tinker A V., Teng NNH, Harrell MI, et al. Secondary Somatic Mutations Restoring RAD51C and RAD51D Associated with Acquired Resistance to the PARP Inhibitor Rucaparib in High-Grade Ovarian Carcinoma. Cancer Discov. 2017;7: 984–998. doi:10.1158/2159-8290.CD-17-0419

104. Ran FA, Hsu PD, Wright J, Agarwala V, Scott DA, Zhang F. Genome engineering using the CRISPR-Cas9 system. Nat Protoc. 2013;8: 2281–2308. doi:10.1038/nprot.2013.143

105. Richardson C, Moynahan ME, Jasin M. Double-strand break repair by interchromosomal recombination: suppression of chromosomal translocations. Genes Dev. 1998;12: 3831–3842. Available: <https://www.ncbi.nlm.nih.gov/pubmed/9869637>

106. Essers J, Hendriks RW, Wesoly J, Beerens CEMT, Smit B, Hoeijmakers JHJ, et al. Analysis of mouse Rad54 expression and its implications for homologous recombination. DNA Repair (Amst). 2002; doi:10.1016/S1568-7864(02)00110-6

113. Martín V, Chahwan C, Gao H, Blais V, Wohlschlegel J, Yates JR, et al. Sws1 is a conserved regulator of homologous recombination in eukaryotic cells. EMBO J. 2006;25: 2564–2574. doi:10.1038/sj.emboj.7601141
