# Supplementary figures and images for: Differential Requirements for the RAD51 Paralogs in Genome Repair and Maintenance in Human Cells
Source: PLoS Genet. 2019 Oct 4;15(10):e1008355. doi: 10.1371/journal.pgen.1008355 (PMC6795472; doi:10.1371/journal.pgen.1008355)

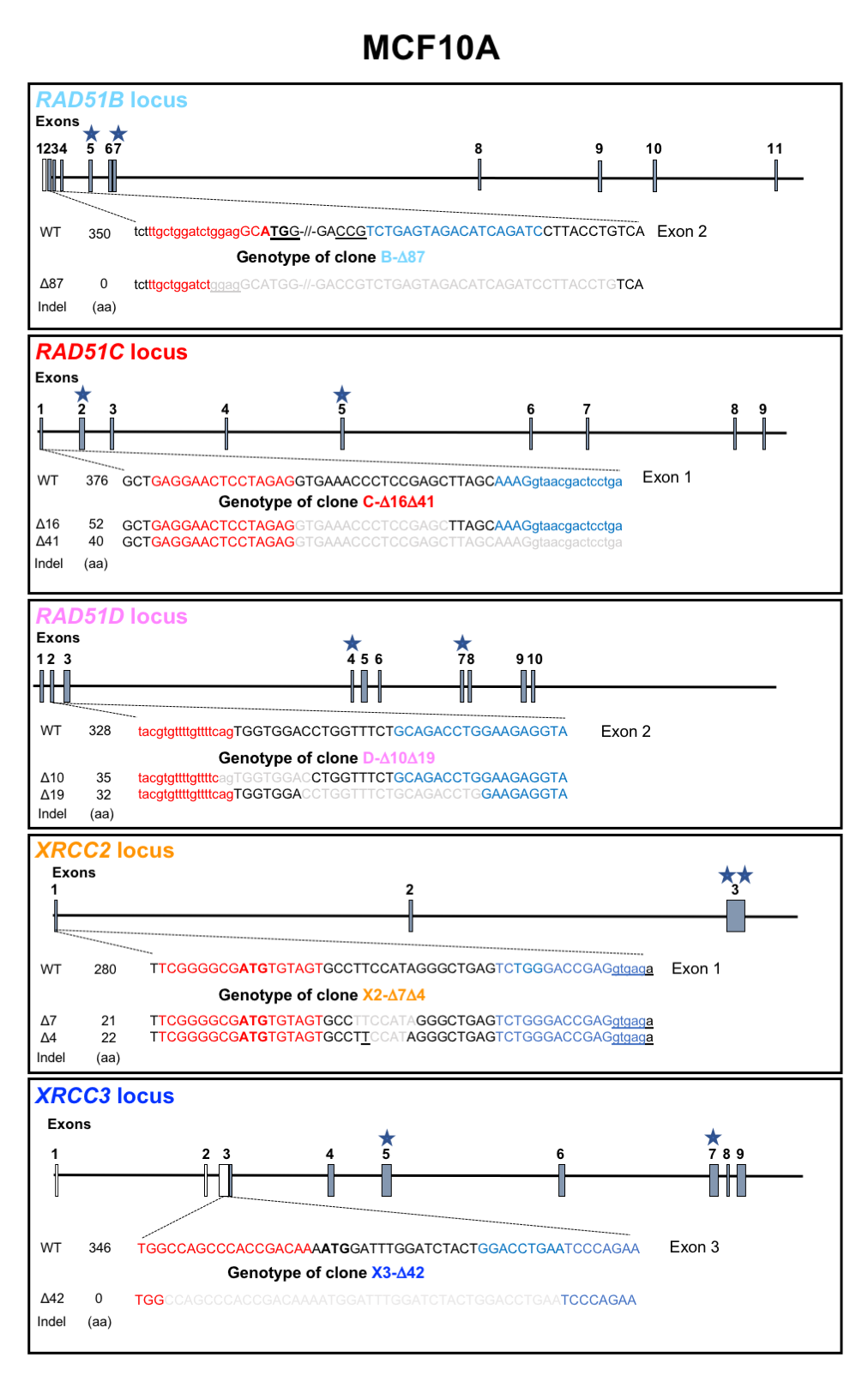

Supplement: S1 Fig — Schematics showing the genomic locus for each RAD51 paralog where filled and clear exons represent coding and non-coding exons respectively (see S1 Table for reference to the Ensembl transcript). Stars indicate the locations of the sequences encoding the Walker A and B motifs. DNA sequence in red and blue indicates the left and right gRNA binding sites and the PAM (underlined) for RAD51B and left and right TALEN recognition sequences for RAD51C, RAD51D, XRCC2 and XRCC3. ATG is shown in bold. Underneath these are the genotypes of the mutant cell lines used in this study with DNA sequences in gray highlighting the indels. (TIF) [file pgen.1008355.s002.tif]

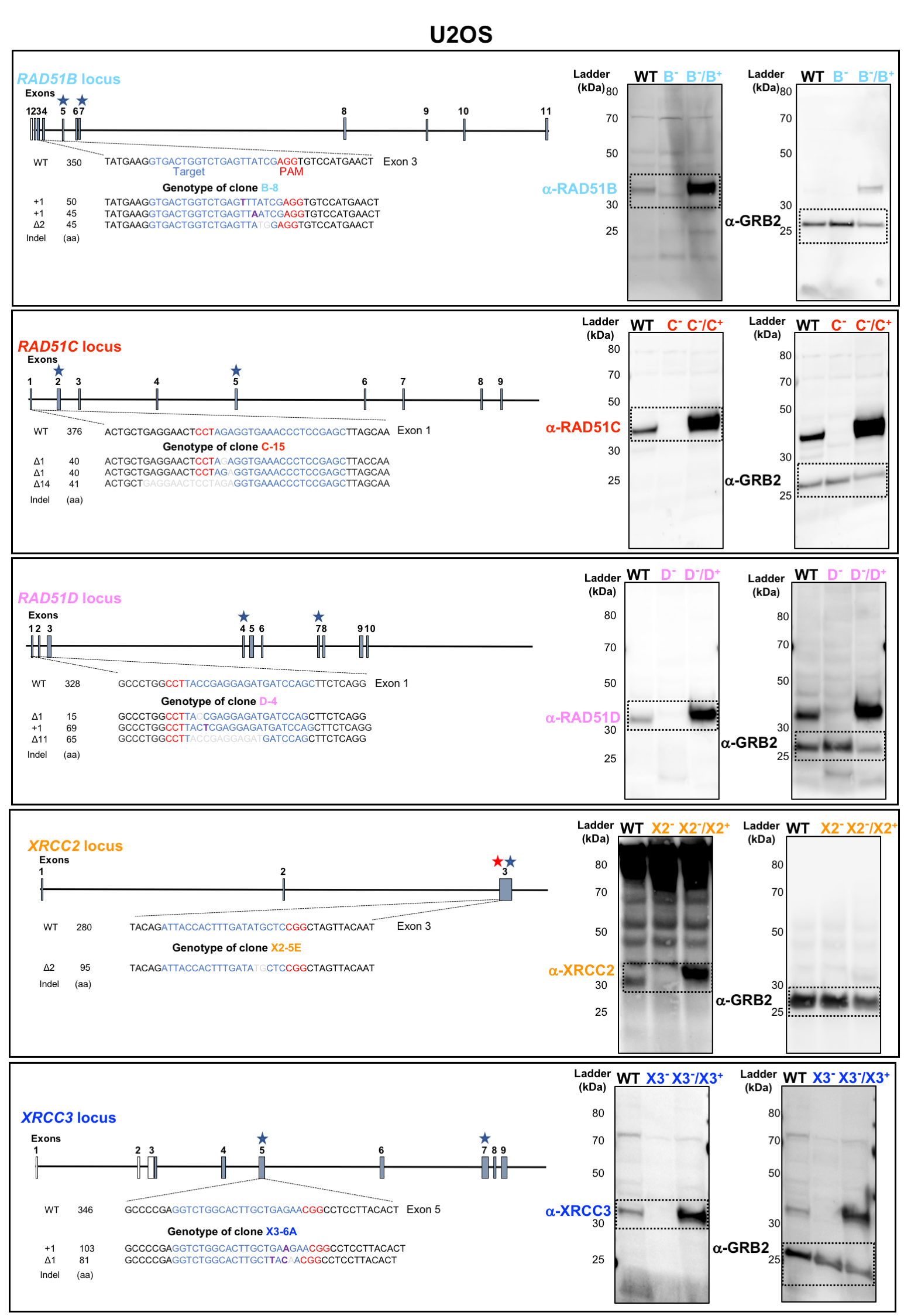

Supplement: S2 Fig — For each RAD51 paralog, a panel is presented to show the organization of the genomic locus (see S1 Table for reference to the Ensembl transcript) and the location of the targeted site by the gRNA (blue) and the PAM (red) early in the coding sequences used for CRISPR-Cas9 genome editing (top). Stars indicate the locations of the sequences encoding the Walker A and B motifs (blue deleted, red retained). The genotype of the mutant cell line with the indels and the size of the predicted truncated polypeptide (a.a.) are shown below. Full-size immunoblots of crude cellular extracts from wild-type cells, mutant cells and mutant cells stably complemented with a retroviral construct expressing the corresponding wild-type allele are shown on the bottom right side. GRB2 was used as loading control. See S1 Table for clone names. (TIF) [file pgen.1008355.s003.tif]

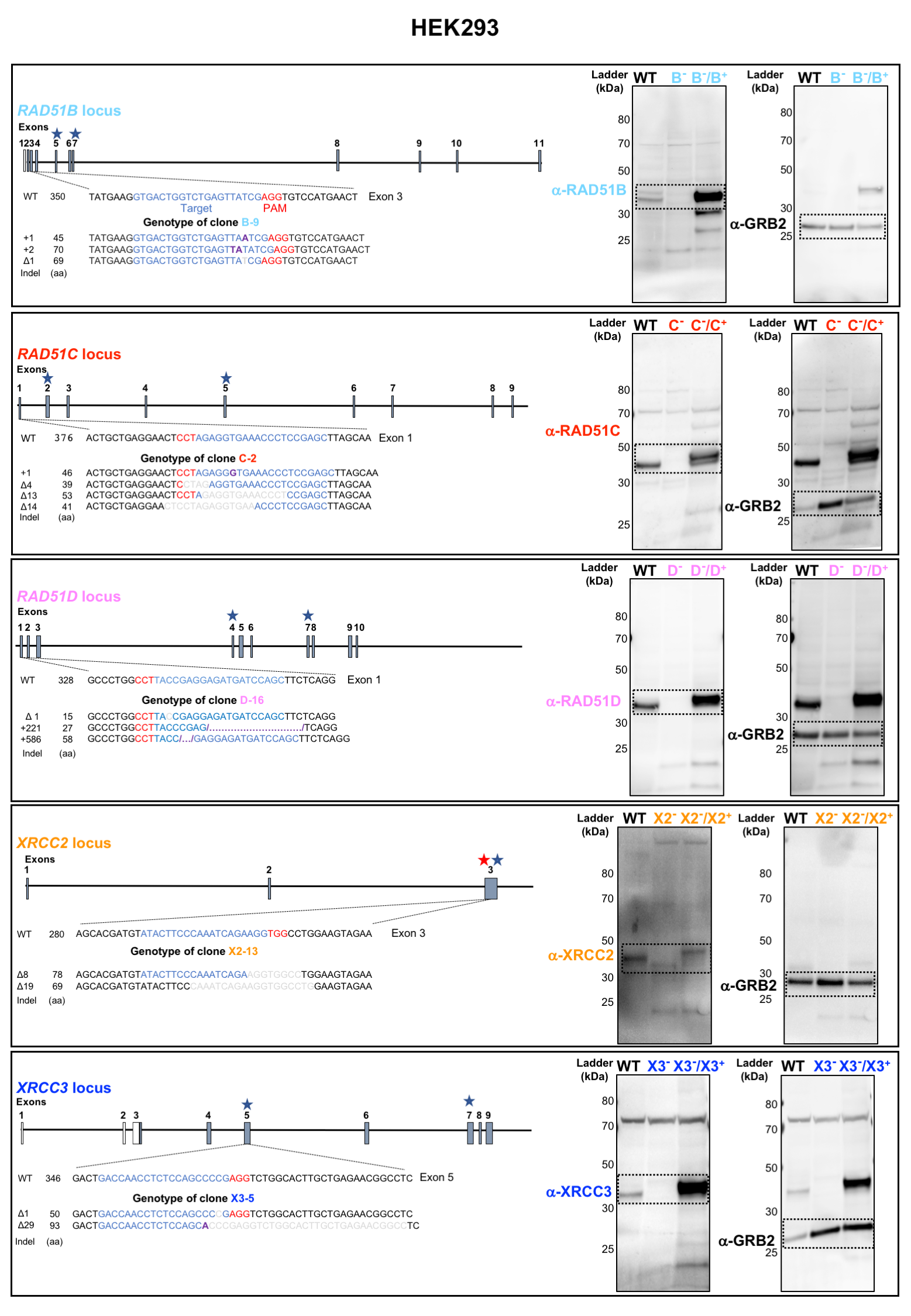

Supplement: S3 Fig — The legend is the same as for S2 Fig. (TIF) [file pgen.1008355.s004.tif]

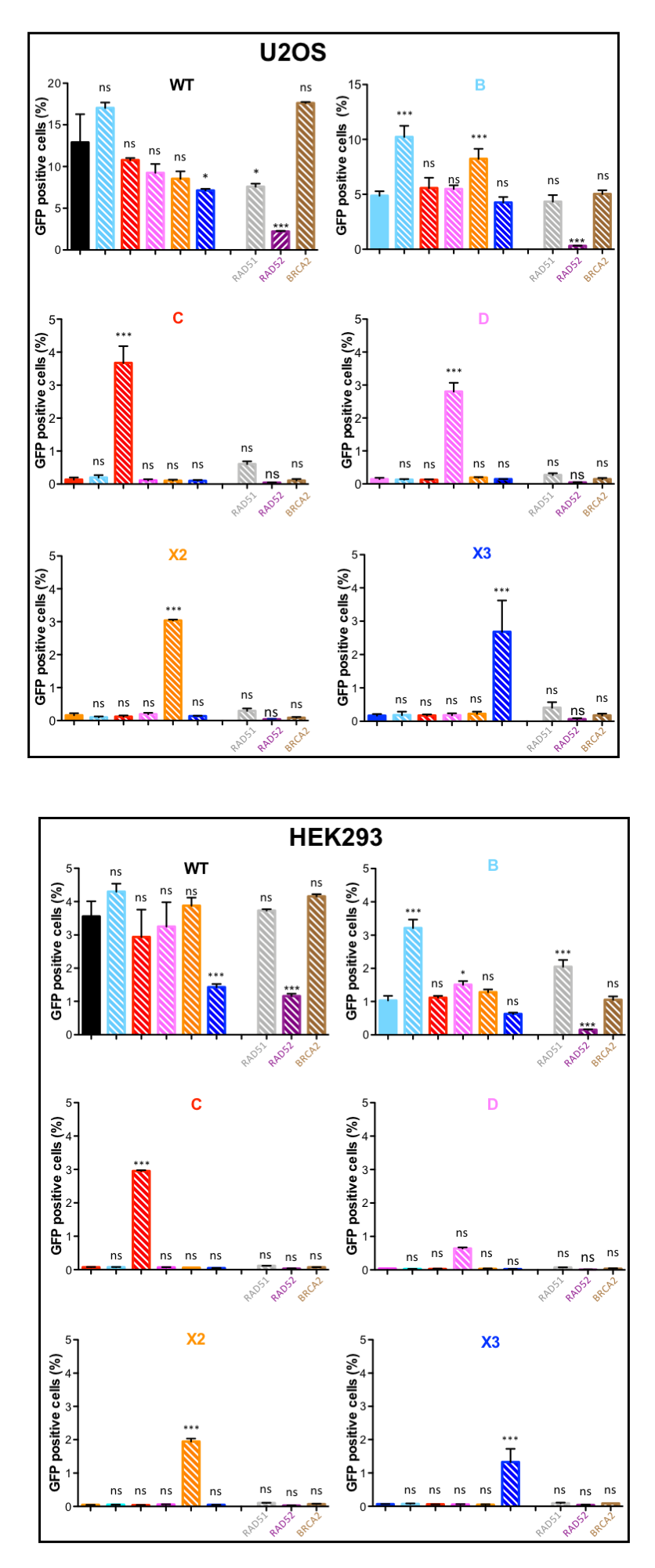

Supplement: S4 Fig — U2OS and HEK293 wild-type and RAD51 paralog mutant cells were transfected with I-SceI-expressing plasmid and with plasmids expressing RAD51 paralogs, RAD51, RAD52 or BRCA2 cDNAs under the control of the strong cytomegalovirus promoter. The frequencies of GFP positive cells were measured 72 h post-transfection. Data are presented as means +/- SD from at least three independent experiments. Differences between complemented cells and either wild-type (WT graph) or mutant cells (B, C, D, X2 and X3) were statistically analyzed using unpaired one-way ANOVA and Tukey's test. * p < 0.05, ** p < 0.01, *** p < 0.001, ns not significant. (TIF) [file pgen.1008355.s005.tif]

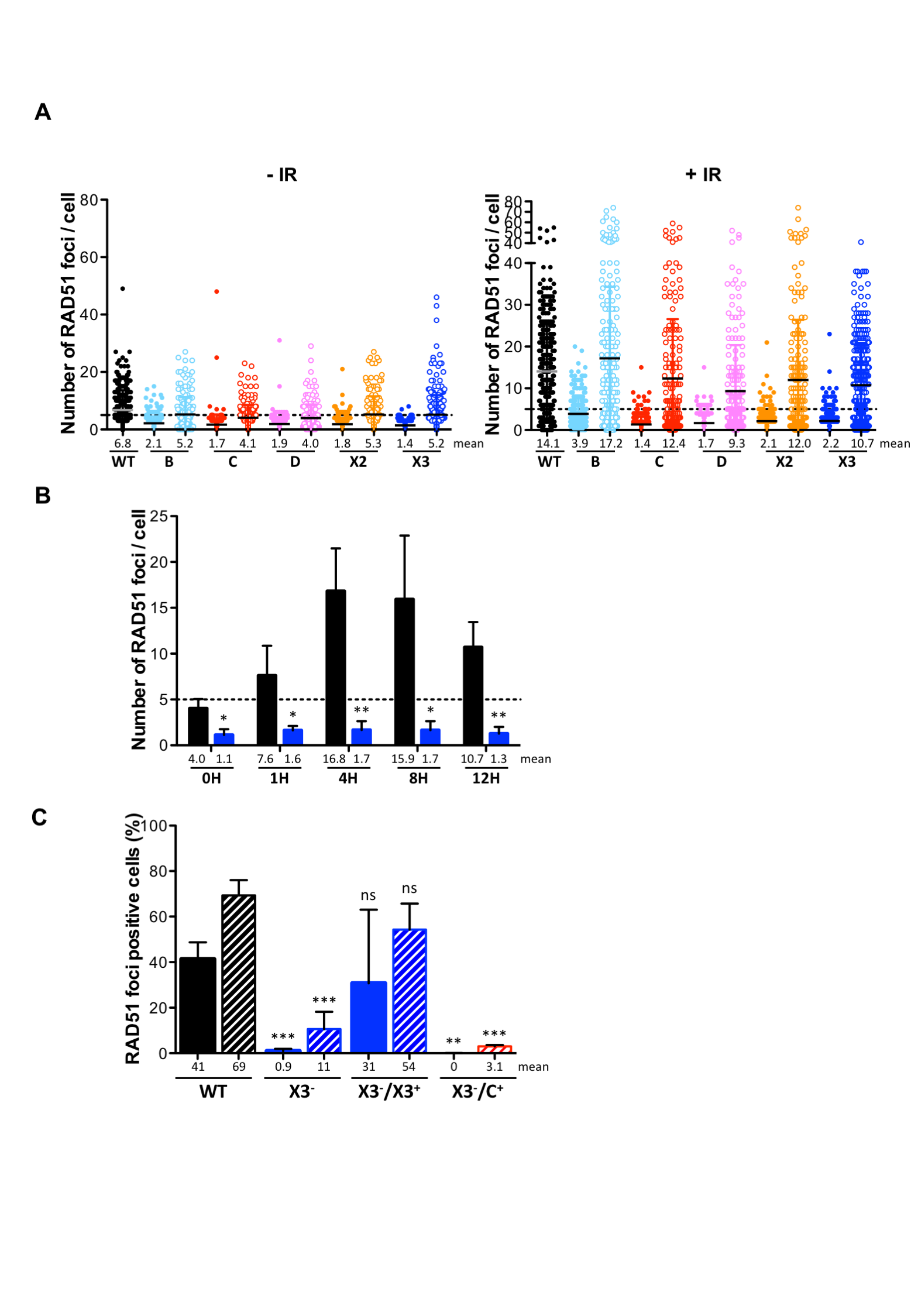

Supplement: S5 Fig — (A) Quantification of RAD51 nuclear focus formation by immunofluorescence in wild-type, RAD51 paralog disrupted and stably complemented RAD51 paralog disrupted U2OS cells exposed to 0 or 4 Gy and after 4 h of recovery. Pool of all the data collected from three experiments for each cell line and condition is presented in the dot plot. Sample sizes from left to right are n = 323, 293, 186, 269, 179, 277, 173, 275, 167, 277, 279 for (-IR) condition; and n = 280, 260, 190, 276, 162, 290, 181, 261, 170, 299, 290 for (+IR) condition. (B) Time course of RAD51 nuclear focus formation by immunofluorescence in wild-type (black) and XRCC3 mutant (blue) U2OS cells exposed to 4 Gy and incubated up to 24 h. At least 100 nuclei were scored for each time point. Results are presented as means +/- SD from three independent experiments. Differences between mutant and wild-type cells were statistically analyzed using unpaired one-way ANOVA and Tukey's test. * p < 0.05, ** p < 0.01. (C) Quantification of RAD51 nuclear focus formation by immunofluorescence in wild-type, XRCC3 mutant, and XRCC3 mutant U2OS cells stably complemented with XRCC3 or RAD51C cDNAs, respectively. Cells were exposed to 0 (filled bars) or 4 Gy (hatched bars) and scored after 4 h recovery. Data for WT, X3 and X3-/X3+ are reported from Fig 4C for comparison to the X3-/C+ experimental samples. In the latter case, two experiments were performed scoring at least 50 nuclei per experiment where in total, 125 and 132 images were analyzed for 0 and 4 Gy conditions, respectively. The data are presented as means +/- SD from the two experiments. Differences between mutant and wild-type cells were statistically analyzed using unpaired T test. ** p < 0.01, *** p < 0.001, ns not significant. Differences between complemented (X3-/X3+ or X3-/C+) and mutant cells (X3-) were all ns in -IR conditions; and, ** and ns for X3-/X3+ and X3-/C+, respectively, in +IR conditions (not indicated in the figure). (TIF) [file pgen.1008355.s006.tif]

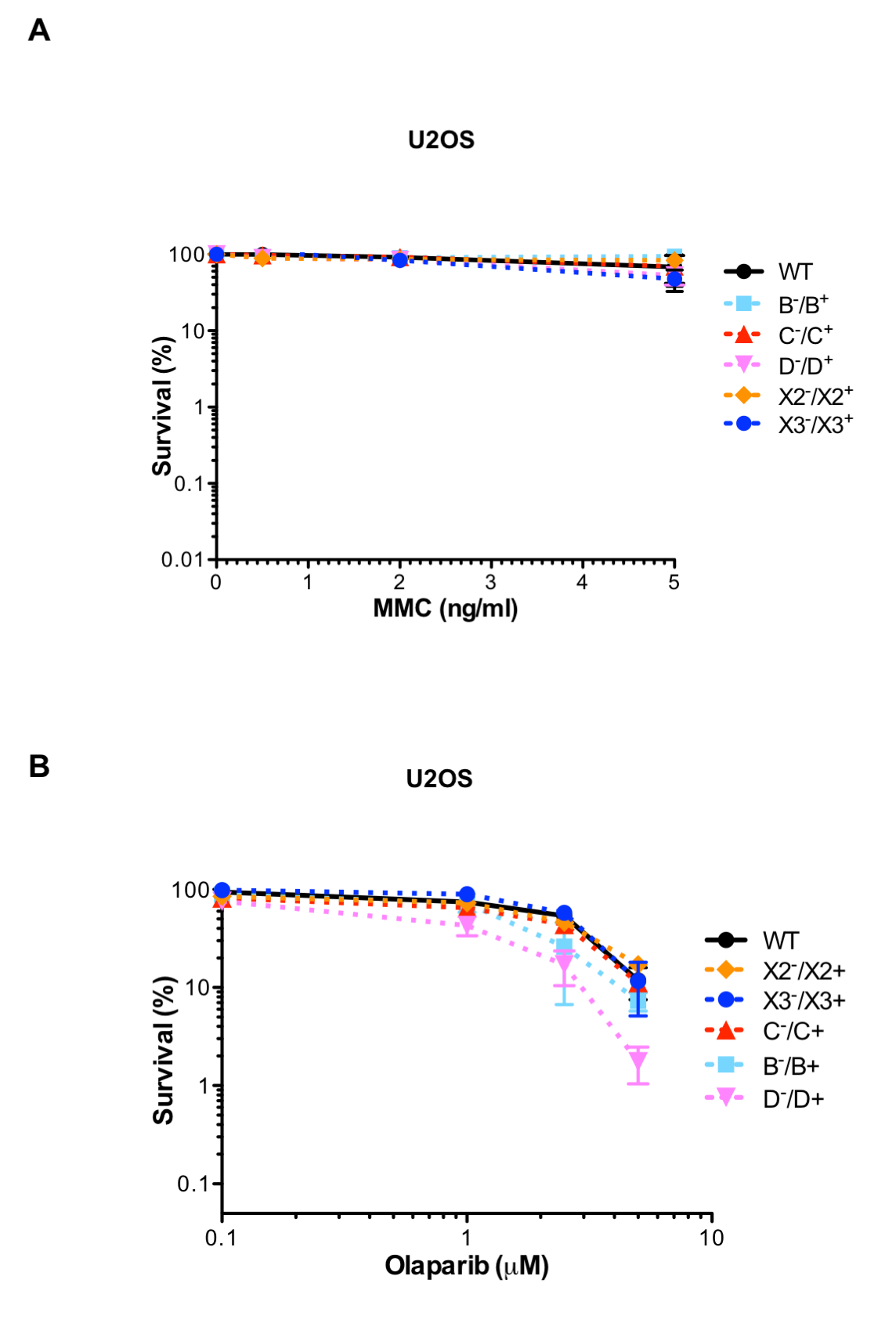

Supplement: S6 Fig — Survival curves obtained by clonogenic cell survival assays after treatment of exponentially growing U2OS cells with indicated doses of (A) mitomycin C (MMC) or (B) olaparib. Analyses of the mutant cells stably complemented with a retroviral construct expressing the corresponding wild-type allele are shown. Results are presented as means +/- SD from at least three independent experiments. These clonogenic survival assays were performed concomitantly with those in main Fig 5. (TIF) [file pgen.1008355.s007.tif]

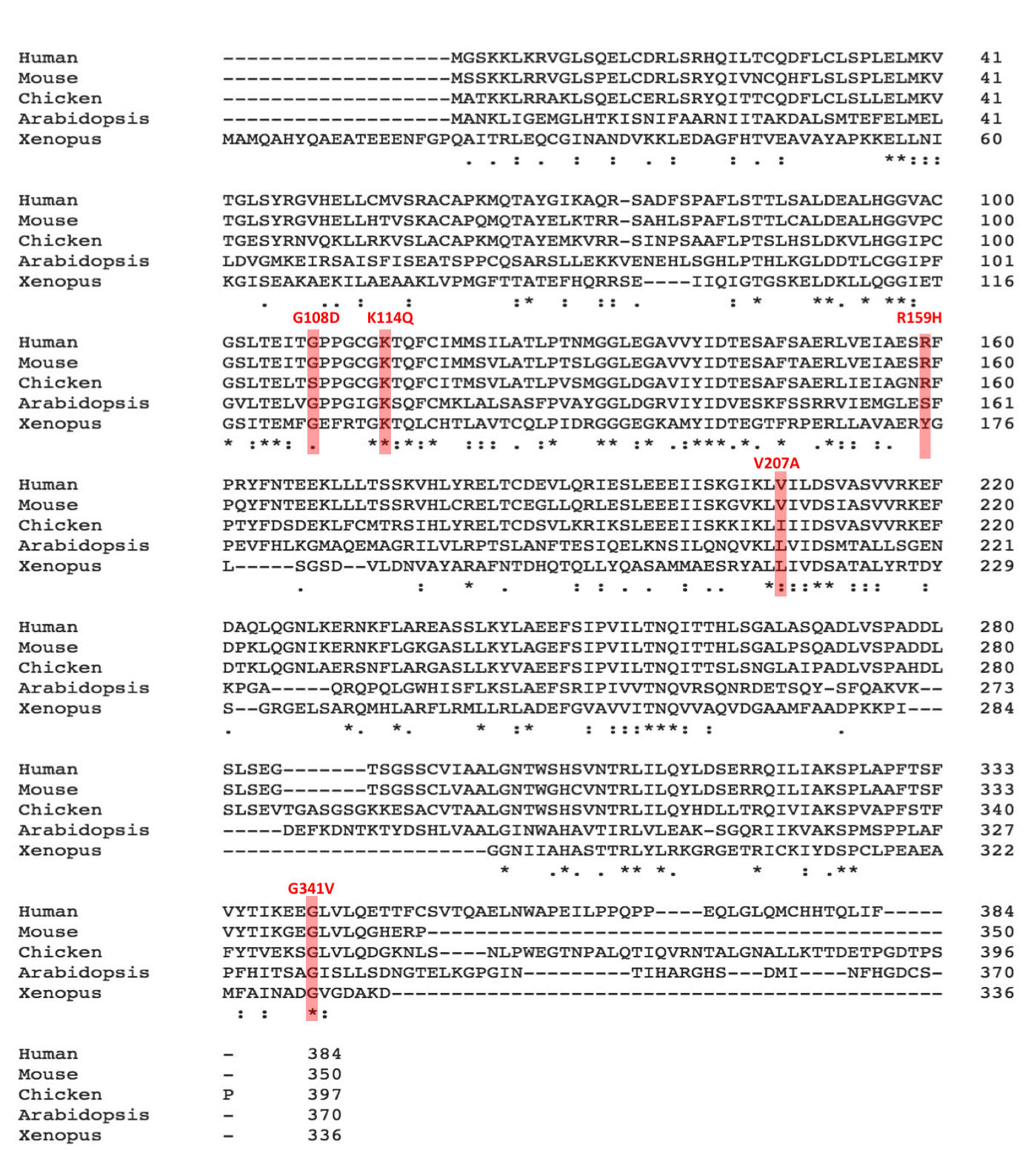

Supplement: S7 Fig — RAD51B point mutations identified in tumors from the MSK-IMPACT database and analyzed in this study (Fig 7) are indicated in red. (TIF) [file pgen.1008355.s008.tif]

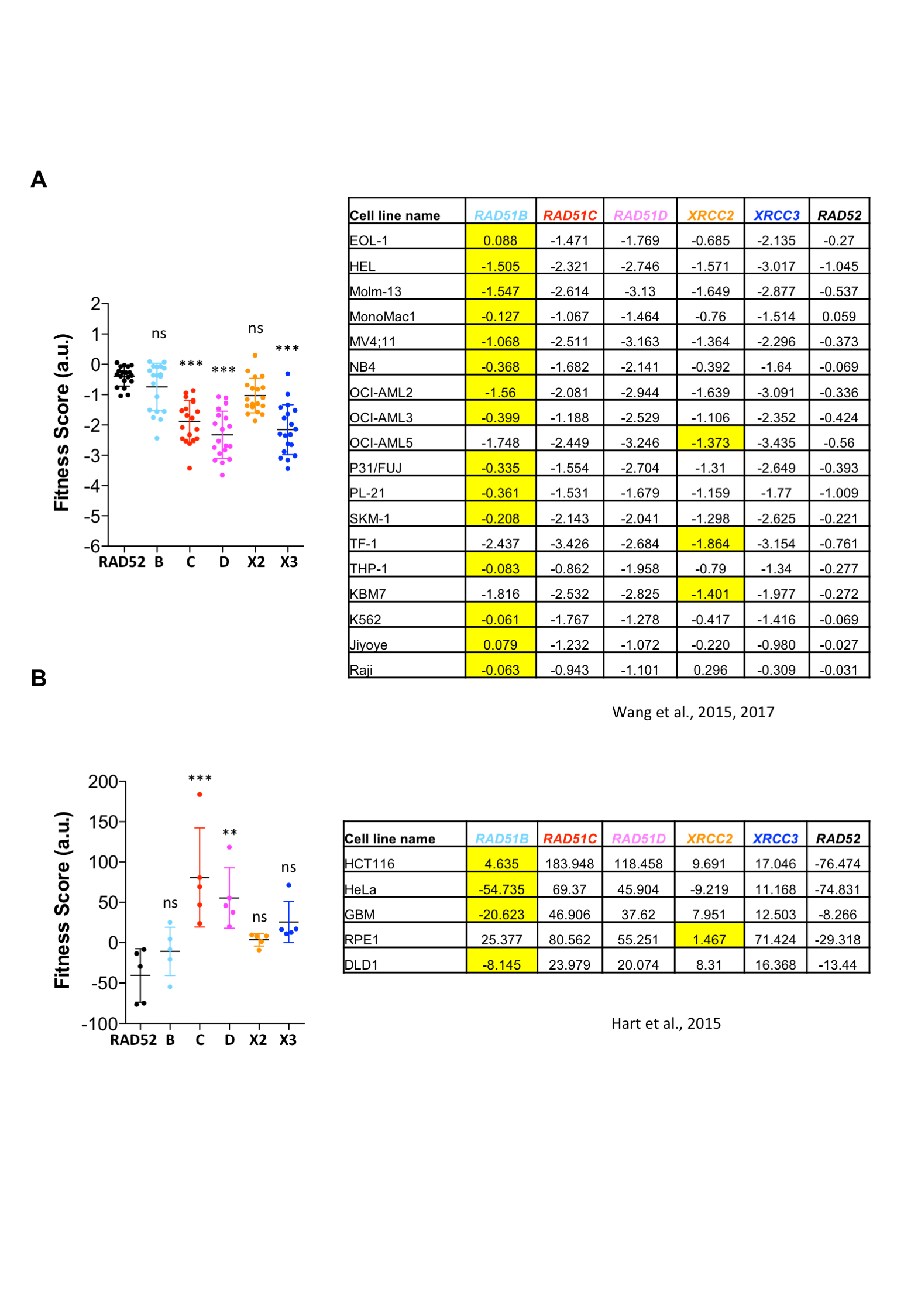

Supplement: S8 Fig — Comparison of fitness scores expressed in arbitrary units from 18 human cell lines [92,93] (A) and 5 human cell lines [91] (B) after RAD52 and RAD51 paralog CRISPR-Cas9 targeting predicts that disruption of RAD51B is more similar to RAD52 disruption than to the disruption of the other RAD51 paralogs in terms of cell survival. Raw CRISPR-Cas9 scores after RAD52 and RAD51 paralogs targeting from various genetic screens are shown on the right of each panel. Yellow highlights indicate the highest relative fitness score. Note that in panel A better fitness tends toward positive numbers but it is reversed in panel B where better fitness tends toward negative numbers. The graphs appear thus as mirror images. Differences between RAD51 paralog mutant and RAD52 mutant cells were statistically analyzed using unpaired one-way ANOVA and Tukey's test. ** p < 0.01, *** p < 0.001, ns not significant. (TIF) [file pgen.1008355.s009.tif]

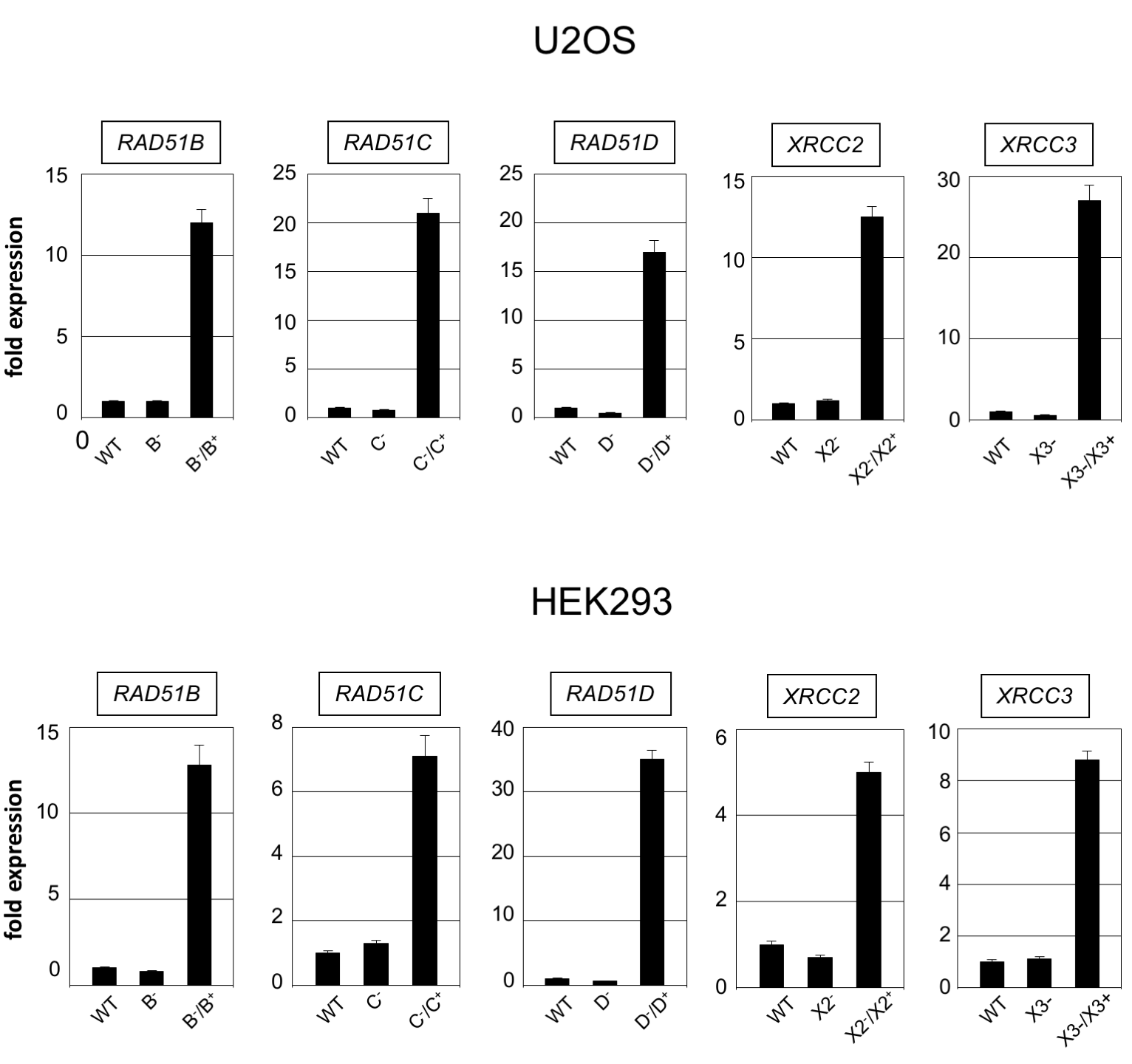

Supplement: S9 Fig — Expression of RAD51B, RAD51C, RAD51D, XRCC2 and XRCC3 was measured by qRT-PCR as indicated in the methods section for wild-type, mutant and complemented mutant cell populations, respectively. Relative expression levels are presented for the U2OS (top) and HEK293 (bottom) cell lines. (TIF) [file pgen.1008355.s010.tif]
