# Supplementary material for: Differential Requirements for the RAD51 Paralogs in Genome Repair and Maintenance in Human Cells
Source: PLoS Genet. 2019 Oct 4;15(10):e1008355. doi: 10.1371/journal.pgen.1008355 (PMC6795472; doi:10.1371/journal.pgen.1008355)
Supplement: S1 Table — (DOCX) [file pgen.1008355.s011.docx]

**S1 Table. Designation of mutant clones.**

|  | Gene | | | | |
| --- | --- | --- | --- | --- | --- |
|  | *RAD51B*  Chromosome 14  Forward strand  Transcript RAD51B-205  ENST00000471583.5 | *RAD51C*  Chromosome 17  Forward strand  Transcript RAD51C-201  ENST00000337432.8 | *RAD51D*  Chromosome 17  Reverse strand  Transcript RAD51D-202  ENST00000345365.10 | *XRCC2*  Chromosome 7  Reverse strand  Transcript XRCC2-201  ENST00000359321.1 | *XRCC3*  Chromosome 14  Reverse strand  Transcript XRCC3-201  ENST00000352127.11 |
| Cell line |  |  |  |  |  |
| MCF10A  (parental) | B1 | C15 | D8.3 | X2-12 | X3-21 |
| MCF10A  (mutant) | B-Δ87 | C-Δ16Δ41 | D-Δ10Δ19 | X2-Δ7Δ4 | X3-Δ42 |
| U2OS | B-8 | C-15 | D-4 | X2-5E | X3-6A |
| HEK293 | B-9 | C-2 | D-16 | X2-13 | X3-5 |
