# Supplementary material for: Differential Requirements for the RAD51 Paralogs in Genome Repair and Maintenance in Human Cells
Source: PLoS Genet. 2019 Oct 4;15(10):e1008355. doi: 10.1371/journal.pgen.1008355 (PMC6795472; doi:10.1371/journal.pgen.1008355)
Supplement: S2 Table — (DOCX) [file pgen.1008355.s012.docx]

**S2 Table. Sequencing results for the genotyping of RAD51 paralog disrupted U2OS cells.**

| **Gene**  **Estimated copy number ***  **Chr #** | **Clone** | **Genomic sequence** (gRNA-PAM) | **Indel (nt)** | **(n)** | **Size**  **(a.a.)** |
| --- | --- | --- | --- | --- | --- |
| RAD51B  6  Chr 14 | **B-8** | CCCCACTGGAGCTTATGAAG**GTGACTGGTCTGAGTTATCGAGG**TGTCCATGAACT | WT | 0 | 350 |
|  |  | CCCCACTGGAGCTTATGAAGGTGACTGGTCTGAGTT**T**ATCGAGGTGTCCATGAACT | +1(T) | 7 | 50 |
|  |  | CCCCACTGGAGCTTATGAAGGTGACTGGTCTGAGTTA**A**TCGAGGTGTCCATGAACT | +1(A) | 13 | 45 |
|  |  | CCCCACTGGAGCTTATGAAGGTGACTGGTCTGAGTTA--GAGGTGTCCATGAACT | -2 | 11 | 45 |
| RAD51C  6  Chr. 17 | **C-15** | TTCCAGACTGCTGAGGAACT**CCTAGAGGTGAAACCCTCCGAGC**TTAGCAAAGGTAACGA | WT | 0 | 376 |
|  |  | TTCCAGACTGCTGAGGAACTCCTA-AGGTGAAACCCTCCGAGCTTACCAAAGGTAACGA | -1(G) | 6 | 40 |
|  |  | TTCCAGACTGCTGAGGAACTCCTAG-GGTGAAACCCTCCGAGCTTAGCAAAGGTAACGA | -1(A) | 6 | 40 |
|  |  | TTCCAGACTGCT--------------GGTGAAACCCTCCGAGCTTAGCAAAGGTAACGA | -14 | 13 | 41 |
| RAD51D  3  Chr. 17 | **D-4** | CGTGCTCAGGGTCGGACTGTGCCCTGG**CCTTACCGAGGAGATGATCCAGC**TTCTCAGG | WT | 0 | 328 |
|  |  | CGTGCTCAGGGTCGGACTGTGCCCTGGCCTTACTCGAGGAGATGATCCAGCTTCTCAGG | +1 | 14 | 69 |
|  |  | CGTGCTCAGGGTCGGACTGTGCCCTGGCCTTA-CGAGGAGATGATCCAGCTTCTCAGG | -1 | 7 | 15 |
|  |  | CGTGCTCAGGGTCGGACTGTGCCCTGGCCTT-----------GATCCAGCTTCTCAGG | -11 | 10 | 65 |
| XRCC2  1  Chr. 7 | **X2-5E** | TTGATACAG**ATTACCACTTTGATATGCTCCGG**CTAGTTACAATTCTTGAGCACAGACTAT | WT | 0 | 280 |
|  |  | TTGATACAGATTACCACTTTGATA—-CTCCGGCTAGTTACAATTCTTGAGCACAGACTAT | -2 | 29 | 95 |
| XRCC3  2  Chr. 14 | **X3-6A** | CCAACCTCTCCAGCCCCGA**GGTCTGGCACTTGCTGAGAACGG**CCTCCTTACACTTGCGG | WT | 0 | 346 |
|  |  | CCAACCTCTCCAGCCCCGAGGTCTGGCACTTGCTGA**A**GAACGGCCTCCTTACACTTGCGG | +1 | 18 | 103 |
|  |  | CCAACCTCTCCAGCCCCGAGGTCTGGCACTTGCTT**AC**-ACGGCCTCCTTACACTTGCGGG | -1 | 10 | 81 |

* retrieved from https://cansar.icr.ac.uk/cansar/cell-lines/U-2-OS/copy_number_variation/
