# Supplementary material for: Differential Requirements for the RAD51 Paralogs in Genome Repair and Maintenance in Human Cells
Source: PLoS Genet. 2019 Oct 4;15(10):e1008355. doi: 10.1371/journal.pgen.1008355 (PMC6795472; doi:10.1371/journal.pgen.1008355)
Supplement: S3 Table — (DOCX) [file pgen.1008355.s013.docx]

| **Gene**  **Estimated copy number ***  **Chr #** | **Clone** | ***Genomic sequence*** *(gRNA-PAM)* | **Indel (nt)** | **(n)** | **Size**  **(a.a.)** |
| --- | --- | --- | --- | --- | --- |
| *RAD51B*    3  Chr. 14 | **B-9** | CCCCACTGGAGCTTATGAAG**GTGACTGGTCTGAGTTATCGAGG**TGTCCATGAACT | WT | 0 | 350 |
|  |  | CCCCACTGGAGCTTATGAAGGTGACTGGTCTGAGTTAATCGAGGTGTCCATGAACT | +1 | 4 | 45 |
|  |  | CCCCACTGGAGCTTATGAAGGTGACTGGTCTGAGTTATATCGAGGTGTCCATGAACT | +2 | 6 | 70 |
|  |  | CCCCACTGGAGCTTATGAAGGTGACTGGTCTGAGTTA-CGAGGTGTCCATGAACT | -1 | 7 | 69 |
| *RAD51C*  4  Chr. 17 | **C-2** | TTCCAGACTGCTGAGGAACT**CCTAGAGGTGAAACCCTCCGAGCTTAGC**AAAGGTAACGA | WT | 0 | 376 |
|  |  | TTCCAGACTGCTGAGGAA--------------ACCCTCCGAGCTTAGCAAAGGTAACGA | -14 | 8 | 41 |
|  |  | TTCCAGACTGCTGAGGAACTCCTA-------------CCGAGCTTAGCAAAGGTAACGA | -13 | 5 | 53 |
|  |  | TTCCAGACTGCTGAGGAACTC----AGGTGAAACCCTCCGAGCTTAGCAAAGGTAACGA | -4 | 14 | 39 |
|  |  | TTCCAGACTGCTGAGGAACTCCTAGAGGGTGAAACCCTCCGAGCTTAGCAAAGGTAACGA | +1 | 12 | 46 |
| *RAD51D*  4  Chr. 17 | **D-16** | CGTGCTCAGGGTCGGACTGTGCCCTGG**CCTTACCGAGGAGATGATCCAGC**TTCTCAGG | WT | 0 | 328 |
|  |  | CGTGCTCAGGGTCGGACTGTGCCCTGGCCTTA-CGAGGAGATGATCCAGCTTCTCAGG | -1 | 17 | 15 |
|  |  | CGTGCTCAGGGTCGGACTGTGCCCTGGCCTTACC**C**GAG/…………………………………/TCAGG | +221 | 11 | 27 |
|  |  | CGTGCTCAGGGTCGGACTGTGCCCTGGCCTTACC/…/CGAGGAGATGATCCAGCTTCTCAGG | +586 | 3 | 58 |
| *XRCC2*  3  Chr. 7 | **X2-13** | TAACAGCACGATGTA**TACTTCCCAAATCAGAAGGTGGCCTGG**AAGTAGAAGTCTTATTTA | WT | 0 | 280 |
|  |  |  |  |  |  |
|  |  | TAACAGCACGATGTATACTTCCCAAATCAGA--------TGGAAGTAGAAGTCTTATTTA | -8 | 15 | 78 |
|  |  | TAACAGCACGATGTATACTTCC-------------------GAAGTAGAAGTCTTATTTA | -19 | 8 | 69 |
| *XRCC3*  3  Chr. 14 | **X3-5** | TGAAGAGACT**GACCAACCTCTCCAGCCCCGAGG**TCTGGCACTTGCTGAGAACGGCCTCCTTA | WT | 0 | 346 |
|  |  |  |  |  |  |
|  |  | TGAAGAGACTGACCAACCTCTCCAGCCC-GAGGTCTGGCACTTGCTGAGAACGGCCTCCTTA | -1 | 12 | 50 |
|  |  | TGAAGAGACTGACCAACCTCTCCAGCA-----------------------------TCCTTA | -29 | 9 | 93 |

**S3 Table. Sequencing results for the genotyping of RAD51 paralog disrupted HEK293 cells.**

* retrieved from http://hek293genome.org/v2/data.php
