# Supplementary material for: Differential Requirements for the RAD51 Paralogs in Genome Repair and Maintenance in Human Cells
Source: PLoS Genet. 2019 Oct 4;15(10):e1008355. doi: 10.1371/journal.pgen.1008355 (PMC6795472; doi:10.1371/journal.pgen.1008355)
Supplement: S4 Table — (DOCX) [file pgen.1008355.s014.docx]

**S4 Table. Genomic PCR primers for MCF10A cells.**

| Gene | Forward primer (5’ to 3’) | Reverse primer (5’ to 3’) | Product size | Restriction enzyme used for genotyping |
| --- | --- | --- | --- | --- |
| *RAD51B* | GTGCCTGTTTGACGAACAATTGTC | GCTGTTACTTCACCTTATGCCGTG | 554 bp |  |
| *RAD51C* | TTTACAAGACTGCGCAAAGCT | AATCTAACGGAGACTGGGCT | 378 bp | BlpI |
| *RAD51D* | GACAGATGAGGAAAAACC | CTGAGAACCCTCTGCTCCCTTTG | 674 bp | SexAI |
| *XRCC2* | GTGGGTGAAAAGTATATCTTTG | AGCAAGTCTTCCAAAGAAAGAC | 1171 bp | EcoNI |
| *XRCC3* | CTTAGCCAGCCAGCGTTTTGTT | TCTCCTCCACCCTCAGTGACAG | 315 bp | BsaBI |
