# Supplementary material for: Differential Requirements for the RAD51 Paralogs in Genome Repair and Maintenance in Human Cells
Source: PLoS Genet. 2019 Oct 4;15(10):e1008355. doi: 10.1371/journal.pgen.1008355 (PMC6795472; doi:10.1371/journal.pgen.1008355)
Supplement: S5 Table — (DOCX) [file pgen.1008355.s015.docx]

**S5 Table. Oligonucleotides for gRNAs targeting RAD51 paralogs.**

| **Gene** | **Upper strand (5’ to 3’)** | **Lower strand (5’ to 3’)** | **Cell line** |
| --- | --- | --- | --- |
| *RAD51B* | CACCGTGACTGGTCTGAGTTATCG | AAACCGATAACTCAGACCAGTCAC | U2OS and HEK293 |
| *RAD51C* | CACCGCTCGGAGGGTTTCACCTCT | AAACAGAGGTGAAACCCTCCGAGC | U2OS and HEK293 |
| *RAD51D* | CACCGCTGGATCATCTCCTCGGTA | AAACTACCGAGGAGATGATCCAGC | U2OS and HEK293 |
| *XRCC2* | CACCGATACTTCCCAAATCAGAAGG | AAACCCTTCTGATTTGGGAAGTATC | HEK293 |
| *XRCC2* | CACCGATTACCACTTTGATATGCTC | AAACGAGCATATCAAAGTGGTAATC | U2OS |
| *XRCC3* | CACCGACCAACCTCTCCAGCCCCG | AAACCGGGGCTGGAGAGGTTGGTC | HEK293 |
| *XRCC3* | CACCGGTCTGGCACTTGCTGAGAA | AAACTTCTCAGCAAGTGCCAGACC | U2OS |

Nucleotides shown in red correspond to the overhangs for subcloning of the annealed oligos into pX458 linearized with BbsI
