# Supplementary material for: Differential Requirements for the RAD51 Paralogs in Genome Repair and Maintenance in Human Cells
Source: PLoS Genet. 2019 Oct 4;15(10):e1008355. doi: 10.1371/journal.pgen.1008355 (PMC6795472; doi:10.1371/journal.pgen.1008355)
Supplement: S6 Table — (DOCX) [file pgen.1008355.s016.docx]

**S6 Table.** **Genomic PCR primers for U2OS and HEK293 cells**

| **Gene** | **Upstream (5’ to 3’)** | **Downstream (5’ to 3’)** |
| --- | --- | --- |
| *RAD51B* | GGTATATGCCAACTAAACAGG | CTGGGCAACAGAGCGTCTCAA |
| *RAD51C* | CTTCCGCTTTACGTCTGACGTCACGC | CTCCTAACCATTCAGACAACTTGTAAG |
| *RAD51D* | GATGACCCCCAGCCCTACCCTTGGTG | CCACCCTTCCTGAGCCTCTCCAGAAG |
| *XRCC2* | GTATTTGCTTGTACAGCTCCATTTTGGC | CACACTTTCTCCTCCATTGACGCGGTC |
| *XRCC3* | TGGTATCTGTCCGAGTGCCAGGAC | TAGGACAAGCAAGATGGGAACTCTG |
