# Supplementary material for: Differential Requirements for the RAD51 Paralogs in Genome Repair and Maintenance in Human Cells
Source: PLoS Genet. 2019 Oct 4;15(10):e1008355. doi: 10.1371/journal.pgen.1008355 (PMC6795472; doi:10.1371/journal.pgen.1008355)
Supplement: S7 Table — (DOCX) [file pgen.1008355.s017.docx]

**S7 Table. Antibodies used in this study**

| Protein | Source | Reference | Dilution for WB or IF | Antigen residues |
| --- | --- | --- | --- | --- |
|  |  |  |  |  |
| RAD51B | SantaCruz | sc-377192 | 1/200 (WB) | Residues 141-255 |
| RAD51C | Abcam | ab95069 | 1/2000 (WB) | Residues 326-376 of hRAD51C (NP_478123.1) |
| RAD51D | Abcam | ab202063 | 1/1000 (WB) | Residues 1-100 |
| XRCC2 | SantaCruz | sc-365854 | 1/200 (WB) | Residues 6-33 |
| XRCC3 | SantaCruz | sc-271714 | 1/200 (WB) | Residues 1-300 |
| GRB2 | BD Bioscience | 610112 | 1/5000 (WB) | Residues 1-217 |
| RAD51 | Roland Kanaar | batch 2307 [106] | 1/5000 (IF) | Full-length RAD51 |
