# Supplementary material for: Differential Requirements for the RAD51 Paralogs in Genome Repair and Maintenance in Human Cells
Source: PLoS Genet. 2019 Oct 4;15(10):e1008355. doi: 10.1371/journal.pgen.1008355 (PMC6795472; doi:10.1371/journal.pgen.1008355)
Supplement: S8 Table — (DOCX) [file pgen.1008355.s018.docx]

**S8 Table. Plasmids used in this study**

| Plasmid trivial name | Lab stock  number | Description/comments | Reference  /source |
| --- | --- | --- | --- |
| pX458 | pMM1242 | Addgene plasmid #48138  AMP^R^ | [104] |
| pX458-RAD51B | pMM1227 | See S5 Table for inserted oligonucleotide sequence  AMP^R^ | This study |
| pX458-RAD51C | pMM1228 | See S5 Table for inserted oligonucleotide sequence  AMP^R^ | This study |
| pX458-RAD51D | pMM1229 | See S5 Table for inserted oligonucleotide sequence  AMP^R^ | This study |
| pX458-XRCC2-#1 | pMM1230 | See S5 Table for inserted oligonucleotide sequence. Used for HEK293.  AMP^R^ | This study |
| pX458-XRCC2-#2 | pMM1532 | See S5 Table for inserted oligonucleotide sequence. Used for U2OS.  AMP^R^ | This study |
| pX458-XRCC3-#1 | pMM1231 | See S5 Table for inserted oligonucleotide sequence. Used for HEK293.  AMP^R^ | This study |
| pX458-XRCC3-#2 | pMM1533 | See S5 Table for inserted oligonucleotide sequence. Used for U2OS.  AMP^R^ | This study |
| pCBA-SceI | pMM1534 | Addgene plasmid # 26477  AMP^R^ | [105] |
| pcDNA-RFP | pMM86 | pcDNA3-TagRFP  AMP^R^ | Evrogen |
| pCMV | pMM99 | pCMV-myc-nuc  AMP^R^ | ThermoFisher |
| pCMV-RAD51B | pMM1222 | RAD51B ORF was cloned using BsmbI/XbaI restriction sites into the NcoI/XbaI sites of pCMV using the following primers:  Forward: AACGTCTCCCatgggtagcaagaaactaaaacgagtg  Reverse: TCTAGACTAGGAATTTCCATAGGC  AMP^R^ | cDNA provided by Roland Kanaar |
| pCMV-RAD51C | pMM1223 | RAD51C ORF was cloned using BsmbI/XbaI restriction sites into the NcoI/XbaI sites of pCMV using the following primers:  Forward: AACGTCTCCCATGCGCGGGAAGACGTTCCGCTTTG  Reverse: TCTAGATTATAATTCTTCCTCTGG  AMP^R^ | cDNA provided by Roland Kanaar |
| pCMV-RAD51D | pMM1224 | RAD51D ORF cloned using BsmbI/XbaI restriction sites into the NcoI/XbaI sites of pCMV using the following primers:  Forward: AACGTCTCCCATGGGCGTGCTCAGGGTCGGACTGTG  Reverse: TCTAGATTATGTCTGATCACCCTG  AMP^R^ | cDNA provided by Roland Kanaar |
| pCMV-XRCC2 | pMM1225 | XRCC2 ORF cloned using BsmbI/XbaI restriction sites into the NcoI/XbaI sites of pCMV using the following primers:  Forward: AACGTCTCCCatgtgtagtgccttccatagggctgag  Reverse: TCTAGATTAACAAAATTCAACCCC  AMP^R^ | cDNA provided by Roland Kanaar |
| pCMV-XRCC3 | pMM1226 | XRCC3 ORF was cloned using BsmbI/XbaI restriction sites into the NcoI/XbaI sites of pCMV using the following primers:  Forward: AACGTCTCCCATGGATTTGGATCTACTGGACCTGAAT  Reverse: TCTAGATTAGTGGGACTGGGTCCC  AMP^R^ | cDNA provided by Roland Kanaar |
| pCMV-RAD51 | pMM1540 | RAD51 ORF was subcloned using NcoI/SalI restriction sites to the NcoI/XbaI sites of pCMV  AMP^R^ | cDNA provided by Roland Kanaar |
| pCMV-RAD52 | pMM1541 | RAD52 ORF was subcloned using NcoI/XhoI restriction sites to the NcoI/XhoI sites of pCMV  AMP^R^ | cDNA provided by Roland Kanaar |
| pCMV-BRCA2 | pMM973 | BRCA2 ORF was subcloned as a NotI/XhoI fragment into the NotI/XhoI sites of a modified pCMV  AMP^R^ | cDNA provided by Kevin Hiom |
| pWZL-RAD51B | pKB506  (pMM1535) | RAD51B ORF was cloned using EcoRI/SalI restriction sites into the pWZL-hygro retroviral vector using the following primers:  Forward: GTGGTGGTACGTAGGAATTCgccaccATGgactacaaagacgatgatgacaagatgggtagcaaga  Reverse: GCGgtcgacCTAGGAATTTCCATAGGCTTGAAGAAC  The pWZL-hygro retroviral vector is Addgene plasmid #18750.  AMP^R^ | This study.  Template cDNA provided by Jun Huang |
| pWZL-RAD51C | pRP6  (pMM1536) | RAD51C ORF was cloned using EcoRI/SalI restriction sites into the pWZL-hygro retroviral vector.  The pWZL-hygro retroviral vector is Addgene plasmid #18750.  AMP^R^ | [103] |
| pWZL-RAD51D | pKB505  (pMM1537) | RAD51D ORF was cloned using EcoRI/SalI restriction sites into the pWZL-hygro retroviral vector using the following primers:  Forward: GTGGTGGTACGTAGGAATTCgccaccATGgactacaaagacgatgatgacaagatgggcgtgctca  Reverse: GCGgtcgacTCATGTCTGATCACCCTGTAATGTG  The pWZL-hygro retroviral vector is Addgene plasmid #18750.  AMP^R^ | This study.  Template cDNA provided by Paul Russell  [113] |
| pWZL-XRCC2 | pKB504  (pMM1538) | XRCC2 ORF was cloned using EcoRI/SalI restriction sites into the pWZL-hygro retroviral vector using the following primers:  Forward: GTGGTGGTACGTAGGAATTCgccaccATGgactacaaagacgatgatgacaagatgtgtagtgccttc  Reverse: GCGgtcgacTCAACAAAATTCAACCCCACTTTCTC  The pWZL-hygro retroviral vector is Addgene plasmid #18750.  AMP^R^ | This study.  Template cDNA provided by Paul Russell  [113] |
| pWZL-XRCC3 | pKB826  (pMM1539) | XRCC3 ORF was cloned using EcoRI/SalI restriction sites into the pWZL-hygro retroviral vector using the following primers:  Forward:vGTGGTGGTACGTAGGAATTCgccaccATGgactacaaagacgatgatgacaagATGGATTTGGATC  Reverse: GCGgtcgacTCAGTGGGACTGGGTCCCAGG  The pWZL-hygro retroviral vector is Addgene plasmid #18750.  AMP^R^ | This study  Template cDNA provided by David Schild  [23] |
| pAAVS1-RAD51B | pRP1 | RAD51B was cloned using EcoRI/SalI restriction sites into the pAAVS1 vector using the following primers:  Forward: CGAAGTTATGAATTCGCCACCATGGGTAGCAAGAAACTAAAACGAG  Reverse: TATACCGGTGTCGACTTAAAAAATTAGCTGGGTATGGTG  pAAVS1 vector is described in [81,83]. | This study |
| pAAVS1-RAD51C | pRP2 | RAD51C was cloned using EcoRI/SalI restriction sites into the pAAVS1 vector using the following primers:  Forward: CGAAGTTATGAATTCGCCACCATGCGCGGGAAGACGTTCC  Reverse: TATACCGGTGTCGACTTATAATTCTTCCTCTGGGTCTCG  pAAVS1 vector is described in [81,83]. | This study |
| pAAVS1-RAD51D | pRP3 | RAD51C was cloned using EcoRI/SalI restriction sites into the pAAVS1 vector using the following primers:  Forward: CGAAGTTATGAATTCGCCACCATGGGCGTGCTCAGGGTCGG  Reverse: TATACCGGTGTCGACTCATGTCTGATCACCCTGTAATGTGG  pAAVS1 vector is described in [81,83]. | This study |
| pAAVS1-XRCC2 | pRP4 | XRCC2 was cloned using EcoRI/SalI restriction sites into the pAAVS1 vector using the following primers:  Forward: CGAAGTTATGAATTCGCCACCATGTGTAGTGCCTTCCATAGG  Reverse: TATACCGGTGTCGACTCAACAAAATTCAACCCCACTTTCTC  pAAVS1 vector is described in [81,83]. | This study |
| pAAVS1- XRCC3 | pRP5 | XRCC3 was cloned using EcoRI/SalI restriction sites into the pAAVS1 vector using the following primers:  Forward: CGAAGTTATGAATTCGCCACCATGGATTTGGATCTACTGGACCTG  Reverse:TATACCGGTGTCGACTCAGTGGGACTGGGTCCCAG  pAAVS1 vector is described in [81,83]. | This study |
| pCMV-RAD51B-G108D | pRP7 | generated by site-directed mutagenesis using pMM1222 as template and the following oligonucleotides:  Upper:5’-CACAGAGATTACAGATCCACCAGGTTGTGG  Lower:5’-CCACAACCTGGTGGATCTGTAATCTCTGTG | This study |
| pCMV-RAD51B-K114Q | pRP8 | generated by site-directed mutagenesis using pMM1222 as template and the following oligonucleotides:  Upper: 5’-CAGGTCCACCAGGTTGTGGACAAACTCAGTTTTGTATAATG  Lower: 5’-CATTATACAAAACTGAGTTTGTCCACAACCTGGTGGACCTG | This study |
| pCMV-RAD51B-R159H | pRP9 | generated by site-directed mutagenesis using pMM1222 as template and the following oligonucleotides:  Upper: 5’-GGTTGAAATAGCAGAATCCCATTTTCCCAGATATTTTAAC  Lower: 5’-GTTAAAATATCTGGGAAAATGGGATTCTGCTATTTCAACC | This study |
| pCMV-RAD51B-V207A | pRP10 | generated by site-directed mutagenesis using pMM1222 as template and the following oligonucleotides:  Upper: 5’-CAAAAGGAATTAAACTTGCGATTCTTGACTCTGTTGC  Lower: 5’-GCAACAGAGTCAAGAATCGCAAGTTTAATTCCTTTTG | This study |
| pCMV-RAD51B-G341V | pRP11 | generated by site-directed mutagenesis using pMM1222 as template and the following oligonucleotides:  Upper: 5’-CATCAAGGAGGAAGTCCTGGTTCTTCAAGC  Lower: 5’-GCTTGAAGAACCAGGACTTCCTCCTTGATG | This study |
| pGAD-C1 | pGAD-C1 | AMP^R^, LEU | [103] |
| pGBD-C1 | pGBD-C1 | AMP^R^, TRP | [103] |
| pGAD-RAD51B | pKB45 | RAD51B cDNA was cloned using EcoRI/SalI restriction sites into the pGAD-C1 vector | [103]  Template cDNA provided by David Schild |
| pGAD-RAD51B-G108D | pKB611 | generated by site-directed mutagenesis using pKB45 as template and the following oligonucleotides:  Upper:5’-CACAGAGATTACAGATCCACCAGGTTGTGG  Lower:5’-CCACAACCTGGTGGATCTGTAATCTCTGTG | This study |
| pGAD-RAD51B-K114Q | pKB612 | generated by site-directed mutagenesis using pKB45 as template and the following oligonucleotides:  Upper: 5’-CAGGTCCACCAGGTTGTGGACAAACTCAGTTTTGTATAATG  Lower: 5’-CATTATACAAAACTGAGTTTGTCCACAACCTGGTGGACCTG | This study |
| pGAD-RAD51B-R159H | pKB613 | generated by site-directed mutagenesis using pKB45 as template and the following oligonucleotides:  Upper: 5’-GGTTGAAATAGCAGAATCCCATTTTCCCAGATATTTTAAC  Lower: 5’-GTTAAAATATCTGGGAAAATGGGATTCTGCTATTTCAACC | This study |
| pGAD-RAD51B-V207A | pKB614 | generated by site-directed mutagenesis using pKB45 as template and the following oligonucleotides:  Upper: 5’-CAAAAGGAATTAAACTTGCGATTCTTGACTCTGTTGC  Lower: 5’-GCAACAGAGTCAAGAATCGCAAGTTTAATTCCTTTTG | This study |
| pGAD-RAD51B-G341V | pKB615 | generated by site-directed mutagenesis using pKB45 as template and the following oligonucleotides:  Upper: 5’-CATCAAGGAGGAAGTCCTGGTTCTTCAAGC  Lower: 5’-GCTTGAAGAACCAGGACTTCCTCCTTGATG | This study |
| pGBD-RAD51C | pKB463 | RAD51C cDNA was cloned using EcoRI/SalI restriction sites into the pGBD-C1 vector | [103]  Template cDNA provided by David Schild |
